# Impact of assistive tool-based training on anxiety, mental focus, and self-confidence among university swimming students

**DOI:** 10.3389/fspor.2026.1788570

**Published:** 2026-05-20

**Authors:** Thekra Alawamleh, Walaa Jumah Alkasasbeh, Bekir Erhan Orhan, Hamzah Alfaouri, Saja Omar Hasan

**Affiliations:** 1 Department of Physical Education, School of Sports Science, The University of Jordan, Amman, Jordan; 2 Faculty of Sports Sciences, Istanbul Aydın University, Istanbul, Türkiye; 3 School of Educational Sciences, The University of Jordan, Amman, Jordan

**Keywords:** anxiety, assistive tools, mental focus, mental health in sport, physical activity, psychological well-being, self-confidence, swimming training

## Abstract

**Introduction:**

The psychological state of university students learning swimming particularly anxiety, mental focus, and self–confidence plays a critical role in skill acquisition and performance outcomes. This study aimed to examine the effects of assistive tool–based training on these psychological variables among beginner female university swimmers.

**Methods:**

A quasi–experimental single–group pre–test–post–test design was employed, involving 60 female students enrolled in a mandatory university swimming course. Participants completed the Athlete Anxiety Questionnaire (AAQ) before and after a three–month structured training program incorporating assistive tools, including kickboards, pull buoys, fins, flotation vests, hand paddles, and a mobile feedback application. The internal consistency of the questionnaire domains was high (Cronbach's alpha = 0.81–0.90). Paired–samples t–tests were conducted to assess pre–post differences, and multivariate analysis of variance (MANOVA) was used to examine the effects of academic year, swimming level, and years of practice.

**Results:**

Assistive tool–based training resulted in significant improvements in self–confidence (pre–test: M = 19.35, SD = 3.80; post–test: M = 21.42, SD = 2.99; *p* = 0.001) and mental focus (pre–test: M = 13.97, SD = 2.70; post–test: M = 14.97, SD = 2.75; *p* = 0.040). However, no significant changes were observed in cognitive anxiety (*p* = 0.372) or somatic anxiety (*p* = 0.297). MANOVA results indicated that academic year had a significant effect on somatic anxiety (F = 7.303, *p* < 0.001), while swimming level and years of practice showed no significant effects on any psychological variables.

**Discussion:**

The findings suggest that assistive tool–based training enhances specific aspects of psychological readiness, particularly self–confidence and mental focus, among beginner swimmers. However, its effects on anxiety appear limited. These results highlight the value of integrating supportive equipment into swimming instruction to facilitate positive psychological and learning outcomes. Given the quasi–experimental design, the findings should be interpreted as indicative of associations rather than causal effects.

## Introduction

Swimming is a complex motor skill that requires the integration of physical, technical, and psychological components for effective performance (1, 2). Among university students learning swimming, psychological factors such as anxiety, mental focus, and self-confidence are particularly important because they can directly influence skill acquisition and performance outcomes (3, 4). Therefore, understanding how these psychological factors are shaped during the instructional process is essential for improving swimming learning.

In this context, athletes' psychological states may be influenced by external factors, particularly training methods and instructional strategies (5). Approaches such as progressive skill-based training (6), individualized feedback (7), and task simplification (8) may support more effective learning experiences. In swimming instruction, assistive tools are also commonly used to modify task demands, reduce physical strain, and help learners focus on specific technical elements (9, 10). These strategies may be especially relevant for beginners, whose learning is often affected by fear, anxiety, and low confidence in the water.

Excessive anxiety may impair motor coordination, attentional control, and learning (11, 12). For example, experimental evidence has shown that anxiety can disrupt attentional control and reduce performance efficiency in motor tasks (13). By contrast, meta-analytic evidence indicates that cognitive and somatic anxiety are only weakly related to sport performance, whereas self-confidence shows the strongest and most consistent association with performance (4). Similarly, more recent systematic review evidence confirms a small but positive confidence–performance relationship (r = 0.25, 95% CI 0.19–0.30) (14). Taken together, these findings suggest that instructional approaches capable of reducing anxiety and enhancing self-confidence may be particularly valuable in swimming learners.

Assistive tools such as kickboards, pull buoys, fins, and flotation devices are frequently used in swimming instruction to support practice and technical development (10, 15). They may improve body positioning, reduce physical demands, and help learners focus on specific movement components (10, 16). Beyond motor learning, these tools may also positively influence psychological states by increasing a sense of control, reducing fear related to water immersion and technical errors, and enhancing motivation (16–19). These effects may be especially relevant for beginners, as reduced anxiety can support attentional focus during skill acquisition, while progressive mastery through assistive tools may strengthen self-confidence (13, 20). In addition, by promoting engagement and positive learning experiences, assistive tools may reinforce both psychological readiness and continued participation in physical activity (21, 22).

Although assistive tools are widely used in swimming instruction, the evidence does not uniformly support their use across all learning contexts (23). Some studies have reported that flotation devices do not necessarily improve aquatic skill learning and may influence movement patterns differently from unsupported practice (24). Moreover, aquatic pedagogy research suggests that skills learned in highly controlled swimming pool environments may not always transfer effectively to other aquatic environments (25, 26). Therefore, the value of assistive tools should be considered in relation to the type of device, the learner's needs, and the specific instructional goal (27).

From a theoretical standpoint, the psychological mechanisms underlying assistive tool-based training can be understood through Cognitive Load Theory (CLT) and Self-Determination Theory (SDT). Cognitive Load Theory (28, 29) suggests that learning becomes more effective when instructional designs reduce unnecessary mental effort, allowing learners to focus on essential task components. In swimming, assistive tools such as buoyancy vests, kickboards, or fins may help simplify task demands and support progressive practice, particularly for beginners (30–32).

Self-Determination Theory (33, 34), in turn, provides a motivational perspective by emphasizing that optimal performance and well-being are supported when the basic psychological needs of autonomy, competence, and relatedness are satisfied. Within swimming instruction, assistive tools and feedback-based strategies may help learners feel more in control of their progress, experience success through manageable challenges, and engage more positively with instructors and peers. Such experiences may foster intrinsic motivation, self-confidence, and sustained mental focus during practice (21, 35). Taken together, CLT and SDT provide a useful theoretical basis for understanding how assistive tools may enhance psychological readiness in beginner swimmers.

Empirical evidence suggests that assistive tools can support both technical and psychological aspects of swimming instruction. By helping learners focus on specific movement components, reducing physical strain, and facilitating task-specific feedback, these tools may improve skill execution while also enhancing concentration, confidence, and engagement during practice (23, 36–40). Such benefits appear particularly important in early learning stages, when fear, anxiety, and difficulty coordinating movements may interfere with skill acquisition (41–43). Overall, these findings are consistent with motor learning principles, which emphasize task simplification, reduced cognitive load, and feedback as key elements for effective skill development (44–46).

Although several studies have examined the impact of assistive tools on technical performance, such as improving body positioning and reducing physical strain (10, 38), research on their effects on psychological variables, namely anxiety, mental focus, and self-confidence among beginners remains limited. For example, Alkasasbeh et al. (2024) found that mobile-assisted swimming applications reduced fear of water and increased motivation, but did not directly assess anxiety, mental focus, or self-confidence (47). Similarly, Sin and Hudayani (2020) reported that swimming boards increased interest in learning freestyle without examining psychological impacts (48). Likewise, van Duijn et al. (2021) studied equipment and environmental influences on aquatic skill learning, yet did not address effects on anxiety, mental focus, or self-confidence (23).

This highlights that, although assistive and technological tools can enhance specific aspects of swimming performance, their effects on psychological variables may vary (49). Moreover, research specifically examining the psychological effects of assistive tool-based training in swimming, particularly among university students, remains limited. Therefore, this study aims to examine the effects of assistive tool-based training on anxiety, mental focus, and self-confidence among beginner university swimming students, while also considering demographic factors such as years of swimming experience, academic year, and current swimming level. Specifically, the study addresses the following research questions: (1) Does assistive tool-based training affect anxiety, mental focus, and self-confidence among beginner university swimming students from pre-test to post-test? and (2) What is the effect of demographic variables (years of swimming experience, academic level, and current swimming level) on these psychological measures when assistive tools are used?

## Methods

### Study design

The present study employed a single-group pre-test–post-test design to examine changes in anxiety, mental focus, and self-confidence among beginner university swimming students following an assistive tool-based training program. The study was conducted over a period of three months, during which participants completed baseline assessments before the intervention and post-intervention assessments after completing the training program. This design was considered appropriate for the present research because it allowed the evaluation of within-group changes in the targeted psychological variables over the course of the intervention.

Although the study did not include a control group, this design was considered appropriate given the educational setting and ethical considerations, as all students were enrolled in a mandatory university swimming course and were required to receive the same instructional program. Therefore, withholding the intervention or creating a non-intervention control group was not feasible. The design allowed for the examination of within-group changes over time; however, the findings should be interpreted with caution, as causal inferences cannot be definitively established.

In terms of ethical considerations, the study protocol was reviewed and approved by the Scientific Research Committee at Al-Ahliyya Amman University (Approval Number: FES-18G-117). In addition, official permission to conduct the study was obtained from the university administration. All participants were informed about the study objectives and procedures and provided written informed consent prior to participation. Participation was entirely voluntary, and students were informed that their decision to participate or withdraw would not affect their academic standing or course grades. All data were collected anonymously, stored securely, and used solely for research purposes in accordance with the Declaration of Helsinki.

### Participants

The study involved a single group of 60 female university students enrolled in a mandatory university swimming course within the Department of Physical Education and Health at Al-Ahliyya Amman University. Participants were recruited using a non-random, non-probability purposive sampling approach, and were selected based on their lack of prior formal swimming training to ensure homogeneity in baseline technical skills. The sample included students from all academic years (first-year, second-year, third-year, and fourth-year), with the following distribution: first-year (*n* = 17), second-year (*n* = 13), third-year (*n* = 12), and fourth-year (*n* = 18).

Although all participants were classified as beginner swimmers in formal swimming, natural variations were observed in their initial swimming proficiency and comfort in water. To ensure a more objective classification of these variations, an initial assessment of swimming skill level was conducted by a swimming instructor using an observational checklist that included fundamental aquatic skills (such as water adaptation, floating ability, breathing control, and coordination). Based on this assessment, participants were categorized into beginner (*n* = 30), intermediate (*n* = 20), and advanced (*n* = 10) levels. These differences were documented to examine the potential influence of baseline skill level on psychological outcomes.

Participant-related characteristics collected for further analysis included years of informal swimming experience, academic year, and current swimming level. Years of informal swimming experience were categorized as less than 1 year (*n* = 25), 1–<3 years (*n* = 20), and more than 3 years (*n* = 15). Inclusion criteria required participants to be female students enrolled in the mandatory swimming course, beginner swimmers with no prior formal training, willing to participate voluntarily and provide informed consent, and able to attend the full three-month training program and complete both pre- and post-test assessments.

Exclusion criteria included prior formal or competitive swimming experience, any medical condition or physical limitation preventing participation, unwillingness to complete the pre- or post-test questionnaires, or withdrawal during the study period. No participants were excluded from the initial sample based on these criteria, and all 60 participants completed the study. All participants voluntarily agreed to participate and provided informed consent.

### Procedure

#### Pre-test phase

At the beginning of the study, data were collected at the start of the academic semester during the first week of the mandatory swimming course, all participants completed the Athlete Anxiety Questionnaire (AAQ) (50) to assess baseline levels of cognitive anxiety, somatic anxiety, mental focus, and self-confidence. The timing of the pre-test ensured that participants had not yet been exposed to structured swimming instruction, minimizing potential learning effects on baseline psychological measures. Following the baseline assessment, participants entered a 12-week structured swimming intervention. The training methods were applied progressively in alignment with participants' developing swimming skill levels, moving from basic water adaptation and safety skills toward more advanced coordination and technique execution.

#### Intervention phase

The intervention lasted 12 weeks, with participants attending three structured swimming sessions per week, each lasting 60 min. During the first two weeks, the focus was on water acclimatization, basic floating, and breathing exercises, using flotation vests and belts to reduce fear and anxiety, with low-intensity exercises emphasizing relaxation and mental focus. This initial stage was intended to enhance water safety, familiarity, and confidence, as supportive flotation devices have been reported to facilitate early aquatic adaptation in beginner learners (23, 51).

In weeks three to six, kickboards and pull buoys were introduced to strengthen leg muscles, improve arm stroke mechanics, and enhance coordination, with moderately challenging exercises and individualized feedback to ensure proper technique and confidence building. Kickboards were used to isolate lower-limb action and body position, whereas pull buoys were used to support arm stroke practice and upper-limb coordination, consistent with previous swimming instruction approaches (51, 52).

Weeks seven to ten focused on skill refinement and psychological enhancement, incorporating fins to improve propulsion and arm-leg coordination, and hand paddles to develop arm strength and stroke precision, while session intensity gradually increased and perceived exertion was monitored to prevent fatigue and maintain focus. The progression of task difficulty and the use of specific assistive tools during this phase were intended to reinforce skill refinement while maintaining psychological comfort and attentional control (23, 53).

During the final two weeks (weeks eleven to twelve), all assistive tools were combined in structured drills to consolidate technical skills, and the mobile application SwimtoFly was used as a supportive instructional aid. The application was used during each training session in the final phase (three times per week) for approximately 10–15 min, where participants viewed instructional videos and received guided feedback on their performance. The role of the application was to supplement in-pool instruction rather than replace it. The application provides step-by-step swimming lessons with visual tutorial content and progress-recording features, and in the present intervention it was used to reinforce technique, support engagement, and assist practice monitoring rather than serve as a standalone intervention (47). In this context, “real-time feedback” refers to immediate feedback provided to participants during or immediately after task execution, either directly from the instructor or supported by the visual demonstrations in the application, allowing participants to correct errors in technique, adjust movement patterns, and improve performance within the same training session.

Exercises were tailored according to participants' progress and comfort in water, and training progression moved stepwise from basic aquatic adaptation to more complex coordination and technique tasks. Swimming performance and technical execution were monitored throughout the intervention by the instructor to ensure safe and appropriate progression.

#### Post-test phase

After completing the 12-week training program, post-test data were collected during the final week of the course under the same conditions as the pre-test, participants again completed the AAQ to evaluate changes in cognitive anxiety, somatic anxiety, mental focus, and self-confidence. The timing of data collection was consistent with the course schedule, and no external academic assessments or unusual conditions were present that could have influenced participants' psychological responses. The pre- and post-test scores were compared to evaluate changes associated with the intervention.

### Instrument and reliability

To assess the psychological variables of interest, namely cognitive anxiety, somatic anxiety, self-confidence, and mental focus, the study employed the Athlete Anxiety Questionnaire (AAQ) developed and validated by Trpkovici et al. (2023) (50). This questionnaire is specifically designed to measure the psychological state of athletes in sports contexts and consists of 20 items distributed across four dimensions: cognitive anxiety (6 items), somatic anxiety (3 items), self-confidence (6 items), and mental focus/concentration (5 items). Participants completed the questionnaire both before (pre-test) and after (post-test) the intervention to evaluate changes resulting from the assistive tool-based training program. Responses were rated on a four-point Likert scale ranging from 1 (Does Not Apply at All) to 4 (Strongly Applies). Negative items were reverse-scored during analysis, and higher scores indicated higher levels of the measured construct within each subscale.

To verify the reliability of the instrument in the present study, it was administered to a pilot sample consisting of 15 students drawn from the study population. Cronbach's alpha coefficient was calculated for each domain as well as for the overall scale. The reliability coefficients ranged from 0.81 to 0.90 across the subscales and reached 0.84 for the overall scale, indicating acceptable to high internal consistency (Table 1). As the Athlete Anxiety Questionnaire (AAQ) is a relatively recent instrument, published applications beyond the original validation study remain limited. However, at least one subsequent study has reported using the AAQ in a sport psychology context, namely Trpkovici et al. (2025) (54).

**Table 1:** Cronbach's alpha coefficients for the questionnaire domains and overall scale.

No.	Domain	Number of items	Cronbach's alpha
1	Cognitive Anxiety	6	0.89
2	Somatic Anxiety	3	0.85
3	Self-Confidence	6	0.81
4	Mental Focus	5	0.90
	Overall Scale	20	0.84

### Response scale and interpretation criteria

To facilitate interpretation of the obtained scores, three levels **(**low, moderate, and high**)** were established for each domain, as shown in Table 2.

**Table 2:** Interpretation criteria for the psychological domains.

Domain	Number of items	Score range	Low	Moderate	High
Cognitive Anxiety	6	6–24	6.0–<12.0	12.0–<18.0	18.0–24.0
Somatic Anxiety	3	3–12	3.0–<6.0	6.0–<9.0	9.0–12.0
Mental Focus	5	5–20	5.0–<10.0	10.0–<15.0	15.0–20.0
Self-Confidence	6	6–24	6.0–<12.0	12.0–<18.0	18.0–24.0

### Statistical analysis

Several statistical methods were used to analyze the study data. First, the reliability of the questionnaire was assessed using Cronbach's Alpha. Second, descriptive statistics, including the mean, standard deviation, and minimum and maximum values, were calculated for each psychological variable before and after the intervention and according to demographic variables. Third, a Paired Samples *t*-test was conducted to compare pre- and post-intervention scores to determine the effect of using assistive training tools on the psychological variables. Finally, Multivariate Analysis of Variance (MANOVA) was employed to examine the impact of demographic variables, including academic year, current swimming level, and years of swimming experience, on the students' psychological variables.

In the present study, the MANOVA was conducted to examine the main effects of these demographic variables on the psychological outcomes. Interaction terms between the demographic factors and the pre–post intervention changes were not included, as the analysis aimed primarily to identify group-level differences rather than moderation effects. Future research with larger and more diverse samples could incorporate interaction models to explore potential moderating roles of demographic characteristics on psychological responses to assistive tool-based training. A *post hoc* power analysis was conducted using GPower 3.1* to evaluate the adequacy of the sample size (*N* = 60). Assuming a medium effect size (Cohen's d = 0.5), an alpha level of 0.05, and a desired statistical power of 0.80, the required minimum sample size was 52 participants. Therefore, the current sample of 60 participants provided sufficient statistical power (1 − *β* = 0.84) to detect medium-sized effects in the paired-samples *t*-tests and MANOVA analyses, supporting the adequacy of the study's statistical design. In addition, an *a priori* sensitivity check indicated that with the same parameters (*α* = 0.05, power = 0.80), the achieved sample size was adequate to detect medium effects for the main outcomes, further supporting the statistical justification for the selected sample size.

## Results

To examine the effect of assistive tool-based training on cognitive anxiety, somatic anxiety, self-confidence, and mental focus among beginner university swimming students, pre-test and post-test means, standard deviations, and paired-samples *t*-test results are presented in Table 3.

**Table 3:** Means, standard deviations, and paired-samples *t*-test results for psychological measures before and after the intervention.

Scale	Pre-test mean (SD)	Post-test mean (SD)	t(df)	p	Cohen's d	95% CI of difference
Cognitive Anxiety	14.84 (5.07)	14.13 (4.91)	0.90 (59)	0.37	0.14	−0.62, 1.40
Somatic Anxiety	6.90 (2.73)	6.42 (2.53)	1.05 (59)	0.30	0.13	−0.49, 1.35
Self-Confidence	19.35 (3.80)	21.42 (2.99)	3.46 (59)	0.001*	0.90	0.75, 3.27
Mental Focus	13.97 (2.70)	14.97 (2.75)	2.09 (59)	0.04*	0.38	0.05, 1.87

* Indicates statistical significance at the 0.05 level (*p* < 0.05).

As shown in Table 3, no statistically significant differences were found between the pre-test and post-test scores for cognitive anxiety [pre-test mean = 14.84 (SD = 5.07), post-test mean = 14.13 (SD = 4.91), t = 0.90, *p* = 0.37, d = 0.14] or somatic anxiety [pre-test mean = 6.90 (SD = 2.73), post-test mean = 6.42 (SD = 2.53), t = 1.05, *p* = 0.30, d = 0.13]. In contrast, self-confidence showed a statistically significant improvement following the intervention [pre-test mean = 19.35 (SD = 3.80), post-test mean = 21.42 (SD = 2.99), t = 3.46, *p* = 0.001, d = 0.90]. Mental focus also improved significantly from pre-test to post-test [pre-test mean = 13.97 (SD = 2.70), post-test mean = 14.97 (SD = 2.75), t = 2.10, *p* = 0.04, d = 0.38]. Overall, these findings indicate that assistive tool-based training was associated with improvements in self-confidence and mental focus, whereas no significant changes were observed in anxiety levels.

Table 4 presents the descriptive statistics of cognitive anxiety, somatic anxiety, self-confidence, and mental focus according to academic year, current swimming level, and years of swimming experience.

**Table 4:** Means and standard deviations of psychological measures by academic year, current swimming level, and years of swimming experience.

Variable	Statistic	Cognitive anxiety	Somatic anxiety	Self-confidence	Mental focus
Academic Year					
First Year	Mean	12.00	9.50	21.00	13.50
	SD	2.45	0.58	2.30	1.00
Second Year	Mean	10.38	5.00	22.00	17.00
	SD	4.50	2.00	2.30	2.73
Third Year	Mean	14.50	5.33	21.60	15.17
	SD	5.03	2.18	3.50	2.94
Fourth Year	Mean	15.27	7.38	21.20	14.38
	SD	4.74	2.42	2.90	2.48
Current Swimming Level					
Beginner	Mean	14.65	6.18	21.29	14.94
	SD	5.43	2.75	2.60	3.01
Intermediate	Mean	13.73	6.77	21.38	14.92
	SD	4.26	2.34	3.52	2.51
Advanced	Mean	10.50	6.00	24.00	16.00
	SD	2.12	0.00	0.00	0.00
Years of Swimming Experience					
Less than 1 year	Mean	14.45	6.45	21.59	14.86
	SD	4.87	2.67	2.60	2.81
1–<3 years	Mean	11.38	5.00	22.25	15.75
	SD	4.07	1.69	2.19	2.43
More than 3 years	Mean	14.90	7.40	20.00	14.80
	SD	5.40	2.07	4.67	2.86

As shown in Table 4, descriptive values were observed across academic year, current swimming level, and years of swimming experience. However, these differences should be interpreted with caution, as the MANOVA results indicated no statistically significant differences between groups. Across academic years, cognitive anxiety, somatic anxiety, self-confidence, and mental focus appeared generally similar across the four groups. According to current swimming level, cognitive anxiety, self-confidence, and mental focus did not show meaningful differences between beginner, intermediate, and advanced swimmers. With regard to years of swimming experience, anxiety levels, self-confidence, and mental focus appeared comparable across the different experience groups. These patterns are descriptive only and should not be interpreted as evidence of true group differences. The statistical significance of group comparisons is presented in Table 5.

**Table 5:** MANOVA results for psychological measures by academic year, current swimming level, and years of swimming practice.

Variable	Measure	F	df	p	Partial *η*^2^
Academic Year	Cognitive Anxiety	2.11	3,56	0.11	0.10
	Somatic Anxiety	7.30	3,56	*p* < 0.001*	0.28
	Self-Confidence	0.19	3,56	0.90	0.01
	Mental Focus	2.36	3,56	0.08	0.11
Current Swimming Level	Cognitive Anxiety	0.46	2,57	0.62	0.01
	Somatic Anxiety	0.94	2,57	0.39	0.03
	Self-Confidence	0.66	2,57	0.51	0.02
	Mental Focus	0.13	2,57	0.87	0.00
Years of Swimming Experience	Cognitive Anxiety	1.43	2,57	0.24	0.04
	Somatic Anxiety	2.10	2,57	0.13	0.06
	Self-Confidence	1.48	2,57	0.23	0.05
	Mental Focus	0.35	2,57	0.70	0.01

* Indicates statistical significance at the 0.05 level (*p* < 0.05).

To further examine whether demographic variables were associated with differences in psychological outcomes, MANOVA results for academic year, current swimming level, and years of swimming practice are presented in Table 5.

As shown in Table 5, academic year had a statistically significant effect only on somatic anxiety (F = 7.30, *p* < 0.001, partial *η*^2^ = 0.28). In contrast, no statistically significant effects of academic year were found for cognitive anxiety, self-confidence, or mental focus (all *p* > 0.05). Moreover, current swimming level did not show statistically significant effects on any of the psychological measures, including cognitive anxiety, somatic anxiety, self-confidence, and mental focus (all *p* > 0.05). Similarly, years of swimming practice was not significantly associated with any of the psychological outcomes (all *p* > 0.05). Overall, these findings indicate that only somatic anxiety differed significantly according to academic year, whereas the remaining psychological measures did not vary significantly by academic year, current swimming level, or years of swimming practice.

## Discussion

The present study aimed to examine the effects of assistive tool-based training on psychological readiness among beginner university swimmers. Overall, the findings suggest that this type of training may be effective in enhancing performance-related psychological factors, particularly self-confidence and mental focus, while having limited impact on deeper emotional states such as cognitive and somatic anxiety.

Self-confidence showed a statistically significant improvement following the intervention, increasing from pre-test to post-test scores, with a large effect size. This indicates a substantial positive change in participants' perceived competence. This finding is consistent with previous research demonstrating that structured and supportive learning environments enhance self-efficacy, especially among novice learners (55, 56). Similarly, studies on beginner swimmers have shown that gradual skill progression and task simplification are associated with increased perceived competence and reduced fear-related barriers, particularly in early learning stages (57–59). In the context of swimming, where fear and uncertainty are common barriers (57), the use of assistive tools such as flotation devices, kickboards, and fins appears to provide a sense of safety and control (60). However, one study suggests that excessive reliance on assistive devices may limit independent skill development and reduce confidence when support is removed (23).

This structured progression likely enabled participants to experience mastery in a gradual and manageable way, reinforcing positive performance perceptions. From a theoretical perspective, these findings can be interpreted through Self-Determination Theory, which highlights the importance of perceived competence in fostering motivation and confidence (61). Therefore, the present findings extend previous literature by demonstrating that assistive tools can enhance confidence specifically among university beginner swimmers, rather than competitive or elite populations (47), and may play a particularly important role during the early stages of skill acquisition by reducing both physical and psychological barriers to learning (51, 60, 62).

Similarly, mental focus demonstrated a statistically significant improvement, although with a moderate effect size (d = 0.38), increasing from pre- to post-intervention scores. This suggests that while the intervention supported attentional processes, its impact was less pronounced compared to self-confidence.

One possible explanation lies in motor learning principles, where reducing task complexity allows learners to allocate attentional resources more effectively (63, 64). Assistive tools enable the decomposition of complex swimming skills into simpler components, facilitating concentration on specific elements such as breathing or coordination (9, 65).

Additionally, the use of real-time feedback through mobile applications may have contributed to improved attentional engagement and error correction (66, 67). This is consistent with research on augmented feedback, which shows that external feedback can enhance focus and motor learning in early stages; however, other studies indicate that excessive reliance on feedback may reduce long-term attentional independence (68, 69). However, the moderate magnitude of change suggests that assistive tools alone may not be sufficient to fully optimize attentional control, and that integrating specific cognitive or attentional training strategies may yield greater improvements.

In contrast, no statistically significant changes were observed in cognitive or somatic anxiety following the intervention. Specifically, cognitive anxiety showed a slight decrease, and somatic anxiety also demonstrated a minor reduction**,** although these changes were not statistically significant.‏ This finding is particularly important, as it highlights a key limitation of assistive tool-based training when used in isolation. While such tools may reduce perceived physical risk and enhance feelings of safety, they do not directly target the cognitive and emotional mechanisms underlying anxiety (70, 71). This result contrasts with studies that have reported significant reductions in anxiety when psychological interventions, such as relaxation training, graded exposure, or other psychological strategies, were integrated into training programs (72–74), particularly among competitive or trained swimmers.

An explanation is that anxiety in beginner swimmers is often deeply rooted in fear of water immersion and loss of control (57, 75), which may require longer intervention durations and more targeted psychological strategies to overcome. In addition, short-term instructional programs that focus primarily on technical skill acquisition may not be sufficient to modify deeper emotional responses, especially when baseline anxiety levels are moderate (76, 77). Furthermore, the relatively short duration of the program and the absence of explicit psychological components may have limited the potential for meaningful emotional adaptation.

Regarding demographic variables, the results indicated that academic year had a significant effect only on somatic anxiety, whereas swimming level and years of experience did not significantly influence psychological outcomes. More specifically, higher somatic anxiety among senior students may be related to increased academic workload, graduation pressure, and career uncertainty, which are common stressors in university populations (78–80). This may suggest that responses to assistive tool-based training are relatively consistent across different levels of prior experience. However, these findings should be interpreted with caution, as subgroup sample sizes may have limited the statistical power to detect smaller effects.

In contrast to athletic populations, where more experienced or elite swimmers tend to report lower anxiety due to repeated exposure and developed coping strategies (81, 82), the present sample consisted of beginner university students, which may explain the absence of such patterns. Additionally, descriptive findings suggested that students with moderate prior exposure showed relatively better psychological profiles compared to absolute beginners and those with unstructured long-term experience, supporting the importance of structured training over mere exposure (83–85). The observed variation in somatic anxiety across academic years may be attributed to external stressors, such as academic demands or future career concerns, rather than the training intervention itself (86–88).

When considered in relation to previous literature, the present findings both support and extend existing knowledge. Consistent with prior studies, assistive tools and structured training environments appear to enhance confidence (89), engagement (47), and skill acquisition (58). However, unlike some studies that reported reductions in anxiety, the current results did not demonstrate similar effects (90).

This discrepancy suggests that assistive tools alone may be insufficient to influence deeper emotional responses unless combined with targeted psychological strategies. Therefore, the present study contributes to the literature by highlighting both the benefits and the limitations of assistive tool-based training, particularly in the context of psychological readiness.

Several limitations should be acknowledged. The use of a single-group pre-test–post-test design limits the ability to draw causal inferences, as external factors may have influenced the observed changes. Additionally, the reliance on self-reported measures may introduce response bias, and the exclusive inclusion of female university students limits the generalizability of the findings. Despite these limitations, the study provides valuable insights into the role of assistive tools in supporting psychological development during early stages of swimming instruction.

In conclusion, the findings suggest that assistive tool-based training may play a meaningful role in enhancing self-confidence and mental focus among beginner swimmers, particularly during early skill acquisition. However, reducing anxiety appears to require more comprehensive approaches that integrate both physical and psychological components. For example, combining assistive tools with relaxation techniques, breathing control exercises, graded exposure to water, and cognitive strategies such as positive self-talk may enhance emotional adaptation and reduce anxiety more effectively. Future research should therefore explore combined interventions that incorporate assistive tools alongside strategies such as relaxation training, mindfulness, and graded exposure to better support emotional adaptation and long-term learning outcomes.

## Conclusion

The present study demonstrates that assistive tool-based training positively influences psychological readiness among beginner university swimmers, particularly by enhancing self-confidence and mental focus. However, no significant changes were observed in cognitive and somatic anxiety, indicating that assistive tools alone may be insufficient to address deeper emotional responses.

These findings suggest that while assistive tools are effective in supporting early skill acquisition and creating a positive learning environment, optimal psychological adaptation may require integrating targeted psychological strategies.

From a practical perspective, assistive tools such as kickboards, pull buoys, fins, and flotation devices primarily enhance motor learning, safety, and technical development, whereas mobile applications and digital feedback systems support motivation, engagement, and individualized instruction. Therefore, combining physical assistive tools with digital feedback approaches may provide a more comprehensive strategy to support both skill development and psychological readiness among beginner swimmers. It should be noted that the present findings are limited to psychological outcomes, and do not necessarily reflect actual improvements in swimming skill or performance level.

### Practical applications

The findings of this study provide several practical implications for swimming instructors, coaches, and physical education practitioners working with beginner swimmers. First, the use of assistive tools such as kickboards, pull buoys, fins, and flotation devices can effectively enhance self-confidence and mental focus during early stages of skill acquisition. These tools help reduce perceived task difficulty and create a supportive learning environment that facilitates progressive skill development.

Second, integrating structured and progressive training programs that incorporate assistive devices may improve learners' engagement and psychological readiness, particularly among individuals who experience fear or low confidence in aquatic environments.

However, as assistive tool-based training alone did not significantly reduce anxiety, it is recommended that instructors combine these tools with psychological strategies such as relaxation techniques, breathing control exercises, and gradual exposure to water to better address emotional responses.

Finally, the inclusion of digital tools, such as mobile applications for feedback and progress monitoring, may further enhance motivation, attentional focus, and learning outcomes in beginner swimming programs.

### Limitations and future research

Several limitations should be acknowledged. First, the study employed a single-group pre-test–post-test design without a control group, which limits the ability to control for potential confounding factors such as individual motor abilities, functional capacities, instructional influences, and environmental conditions. Therefore, the observed changes should be interpreted as associative rather than causal. Second, the sample included only female university students, reducing generalizability to male students or other age groups. Third, the study focused on short-term effects over a three-month period, and long-term psychological outcomes were not assessed. Fourth, the study relied primarily on self-report questionnaires to measure psychological outcomes, without incorporating formal objective performance or skill metrics, which limits the ability to substantiate effectiveness claims of the intervention. Fifth, the distribution of participants across academic years and swimming levels was not fully balanced, with some subgroups including fewer than 15 participants. Although this did not affect the main statistical analyses, it may have reduced the sensitivity to detect potential differences between groups. Future research should consider experimental or quasi-experimental designs that include a control or comparison group to strengthen causal inferences and reduce potential bias. It is also recommended to include mixed-gender and more diverse samples to explore possible gender-related or contextual differences in psychological responses. Additionally, long-term follow-up assessments are needed to examine the persistence and stability of improvements in confidence, focus, and anxiety reduction over time. Future investigations could also apply this framework to other sport domains such as gymnastics, martial arts, or team sports to assess whether assistive tool-based training yields similar psychological and technical benefits beyond swimming.

Incorporating objective performance-based measures such as timed swims, technical skill evaluations, or wearable sensor data can provide more robust evidence of the intervention's effectiveness. Furthermore, exploring the integration of emerging technologies, including virtual or augmented reality, may further enhance understanding of how assistive tools influence both skill acquisition and psychological readiness in swimming and other physical education contexts.

Regarding measurement validity, the Anxiety Assessment Questionnaire (AAQ) was selected because of its established psychometric reliability and frequent use in sports psychology research. However, it was originally developed for competitive or trained athlete populations, which may limit its sensitivity in detecting short-term psychological changes among beginner, non-athlete participants. Therefore, the non-significant reduction in anxiety observed in this study may partly reflect the instrument's limited responsiveness rather than the absence of actual psychological improvement. Future research could employ or adapt measurement tools specifically designed for novice or recreational populations to enhance sensitivity to short-term emotional changes.

Additionally, potential expectancy effects should be acknowledged, as participants might have experienced perceived improvements in confidence or focus simply due to their awareness of participating in a structured intervention using assistive tools. This expectancy bias may have influenced self-reported outcomes. Moreover, the study did not include longitudinal tracking after the intervention, which limits understanding of whether the observed psychological changes were sustained over time. Future research incorporating long-term follow-up measurements would provide a clearer picture of the persistence and stability of these effects.

## Data Availability

The raw data supporting the conclusions of this article will be made available by the authors, without undue reservation.
